# Poly[bis­[μ-1,4-bis­(imidazol-1-ylmeth­yl)benzene]­dichloridomanganese(II)]

**DOI:** 10.1107/S1600536811029485

**Published:** 2011-07-30

**Authors:** Chong-Zhen Mei, Wen-Wen Shan, Kai-Hui Li

**Affiliations:** a North China University of Water Conservancy and Electric Power, Zhengzhou 450011, People’s Republic of China

## Abstract

In the crystal structure of the title compound, [MnCl_2_(C_14_H_14_N_4_)_2_]_*n*_, the Mn^II^ atom, lying on an inversion center, is coordinated by four N atoms from four 1,4-bis­(imidazol-1-ylmeth­yl)benzene (bimb) ligands and two Cl^−^ anions in a distorted octa­hedral geometry. The bimb ligands bridge the Mn^II^ atoms, forming a two-dimensional polymeric complex, which is composed of a 52-membered [Mn_4_(bimb)_4_] ring with distances of 7.7812 (2) and 27.4731 (9) Å between opposite metal atoms. Weak C—H⋯π inter­actions are present in the crystal structure.

## Related literature

For the background to the network topologies and applications of coordination polymers, see: Maspoch *et al.* (2007 ▶); Ockwig *et al.* (2005 ▶); Zang *et al.* (2006 ▶); Zhang *et al.* (2009 ▶). For related syntheses and structures of compounds with a bimb ligand, see: Hoskins *et al.* (1997 ▶).
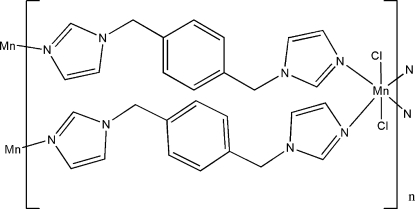


## Experimental

### 

#### Crystal data

[MnCl_2_(C_14_H_14_N_4_)_2_]
*M* *_r_* = 602.42
Monoclinic, 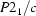

*a* = 7.7812 (2) Å
*b* = 12.7910 (3) Å
*c* = 14.2575 (4) Å
β = 105.539 (3)°
*V* = 1367.17 (6) Å^3^
*Z* = 2
Mo *K*α radiation
μ = 0.71 mm^−1^
*T* = 296 K
0.21 × 0.20 × 0.19 mm

#### Data collection

Bruker SMART APEXII CCD area-detector diffractometer
Absorption correction: multi-scan (*SADABS*; Bruker, 2005 ▶) *T*_min_ = 0.865, *T*_max_ = 0.877
3921 measured reflections
2367 independent reflections
2102 reflections with *I* > 2σ(*I*)
*R*_int_ = 0.015

#### Refinement

*R*[*F* ^2^ > 2σ(*F* ^2^)] = 0.028
*wR*(*F* ^2^) = 0.093
*S* = 1.04
2367 reflections
178 parameters
H-atom parameters constrained
Δρ_max_ = 0.24 e Å^−3^
Δρ_min_ = −0.25 e Å^−3^

### 

Data collection: *APEX2* (Bruker, 2005 ▶); cell refinement: *SAINT* (Bruker, 2005 ▶); data reduction: *SAINT*; program(s) used to solve structure: *SHELXTL* (Sheldrick, 2008 ▶); program(s) used to refine structure: *SHELXTL*; molecular graphics: *DIAMOND* (Brandenburg, 2010 ▶); software used to prepare material for publication: *SHELXTL*.

## Supplementary Material

Crystal structure: contains datablock(s) I, global. DOI: 10.1107/S1600536811029485/xu5253sup1.cif

Structure factors: contains datablock(s) I. DOI: 10.1107/S1600536811029485/xu5253Isup2.hkl

Additional supplementary materials:  crystallographic information; 3D view; checkCIF report

## Figures and Tables

**Table 1:** Selected bond lengths (Å)

Mn1—N1	2.2695 (13)
Mn1—N3	2.2665 (14)
Mn1—Cl1	2.5639 (4)

**Table 2:** Hydrogen-bond geometry (Å, °) *Cg* is the centroid of the N3,N4,C8–C10 ring.

*D*—H⋯*A*	*D*—H	H⋯*A*	*D*⋯*A*	*D*—H⋯*A*
C4—H4*A*⋯*Cg*^i^	0.97	2.65	3.522 (2)	150

Symmetry code: (i) 
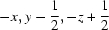
.

**Fig. 1.**
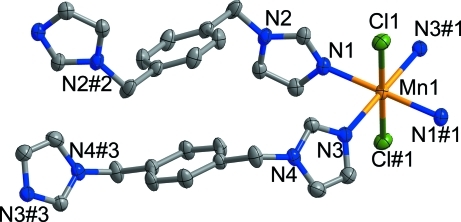
Metal coordination and atom labeling in title compound (thermal ellipsoids at 50% probability level). All hydrogen atoms are omitted for clarity.

**Fig. 2.**
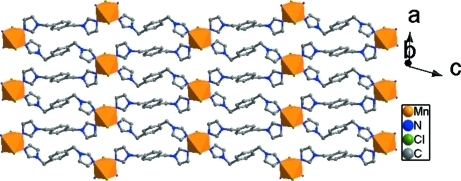
A view of the layer structure in compound 1. Mn atoms are drawn as polyhedrons.

## supplementary crystallographic information

### Comment

The supramolecular coordination assemblies are of great interest not only for their variety of architectures but also for the potential applications as functional materials (Maspoch *et al.*, 2007; Ockwig *et al.*, 2005). Many nitrogen-containing ligands have been successfully employed in the construction of the coordination compounds due to they can satisfy and even mediate the coordination needs of the metal center and consequently generate more meaningful architectures, in which supramolecular contacts (hydrogen bonding, π-π stacking) frequently occur (Zang *et al.*, 2006; Zhang *et al.* 2009). To further explore various factors that influence the formation of result structures in the assembly reactions, we undertake synthetic and structural studies on one novel Mn(II) coordination polymers based on the highly flexible bidentate ligand 1,4-bis(imidazol-1-ylmethyl)-benzene (1,4-bimb): [Mn(bimb)_2_Cl_2_]_n_).

The metal-ligand connectivity pattern of complex is depicted in Figure 1. There are one kind of Mn(II) ion, one kind of Cl^-^ and two kinds of bimb ligands in the structure. Metal center displays a symmetrical Cl_2_N_4_ octahedral geometry, and the related bond distances and bond angles are all symmetrically equivalent. Four N atoms from different bimb ligands comprise the equatorial plane, while two Cl^-^ occupy the axial positions. Each bimb ligand acts as a *µ*_2_-bridge in *trans-*conformation with the planes of the two imidazole rings parallel. As shown in Figure 2, two rows of Mn(II) cations are linked together through bimb ligands to form a *meso*-helix running along the *a*-axis. Adjacent *meso*-helixes are associated together by sharing metal ions to form a two-dimensional architecture, in which large 52-membered rings [Mn_4_(bimb)_4_] with the opposite Mn···Mn distances being 7.7812 (2) Å and 27.4731 (9) Å are detected. If the metal center is considered as a four-connected node, the individual two-dimensional network can be described as a (4,4)-net. Further investigation shows that C8—H8···Cl1^i^ hydrogen bonding is contribute to the stability of the layer. Neighboring layers are arranged parallel with the coordinated Cl^-^ closed to H2 atoms of imidazole ring from adjacent layer, and interlayer C2—H2···Cl1^ii^ hydrogen bonds can be detected which lead to the formation of the three-dimensional supromolecular structure, as shown in Figure 3. The hydrogen-bonding geometry is listed in Table 1.

### Experimental

1,4-Bis(imidazol-1-ylmethyl)-benzene (bimb) was prepared according to the literature (Hoskins *et al.*, 1997), all other starting materials were of analytical grade and obtained from commercial sources without further purification. The title compound was synthesized hydrothermally in a Teflon-lined stainless steel container by heating a mixture of 1,4-bis(imidazol-1-ylmethyl)-benzene (bimb) (0.0119 g, 0.05 mmol), MnCl_2_.4H_2_O (0.0099 g, 0.05 mmol) and NaOH (0.0040 g, 0.1 mmol) in 7 ml of distilled water at 120°C for 3 days, and then cooled to room temperature. Yellow block crystals were obtained in 68% yield based on manganese.

### Refinement

H atoms were positioned geometrically and refined using a riding model, with C—H = 0.93 Å, *U*_iso_(H) = 1.2U_eq_(C) for aromatic H, and C—H = 0.97 Å, *U*_iso_(H) = 1.2U_eq_(C) for CH_2_.

### Crystal data

**Table:**

[MnCl_2_(C_14_H_14_N_4_)_2_]	*F*(000) = 622
*M**_r_* = 602.42	*D*_x_ = 1.463 Mg m^−^^3^
Monoclinic, *P*2_1_/*c*	Mo *K*α radiation, λ = 0.71073 Å
Hall symbol: -P 2ybc	Cell parameters from 2678 reflections
*a* = 7.7812 (2) Å	θ = 3.0–25.1°
*b* = 12.7910 (3) Å	µ = 0.71 mm^−^^1^
*c* = 14.2575 (4) Å	*T* = 296 K
β = 105.539 (3)°	Block, yellow
*V* = 1367.17 (6) Å^3^	0.21 × 0.20 × 0.19 mm
*Z* = 2	

### Data collection

**Table:**

Bruker SMART APEXII CCD area-detector diffractometer	2367 independent reflections
Radiation source: fine-focus sealed tube	2102 reflections with *I* > 2σ(*I*)
graphite	*R*_int_ = 0.015
ω scans	θ_max_ = 25.0°, θ_min_ = 3.0°
Absorption correction: multi-scan (*SADABS*; Bruker, 2005)	*h* = −9→9
*T*_min_ = 0.865, *T*_max_ = 0.877	*k* = −7→15
3921 measured reflections	*l* = −16→10

### Refinement

**Table:**

Refinement on *F*^2^	Primary atom site location: structure-invariant direct methods
Least-squares matrix: full	Secondary atom site location: difference Fourier map
*R*[*F*^2^ > 2σ(*F*^2^)] = 0.028	Hydrogen site location: inferred from neighbouring sites
*wR*(*F*^2^) = 0.093	H-atom parameters constrained
*S* = 1.04	*w* = 1/[σ^2^(*F*_o_^2^) + (0.069*P*)^2^] where *P* = (*F*_o_^2^ + 2*F*_c_^2^)/3
2367 reflections	(Δ/σ)_max_ < 0.001
178 parameters	Δρ_max_ = 0.24 e Å^−^^3^
0 restraints	Δρ_min_ = −0.25 e Å^−^^3^

### Special details

**Table:**

Geometry. All e.s.d.'s (except the e.s.d. in the dihedral angle between two l.s. planes) are estimated using the full covariance matrix. The cell e.s.d.'s are taken into account individually in the estimation of e.s.d.'s in distances, angles and torsion angles; correlations between e.s.d.'s in cell parameters are only used when they are defined by crystal symmetry. An approximate (isotropic) treatment of cell e.s.d.'s is used for estimating e.s.d.'s involving l.s. planes.
Refinement. Refinement of *F*^2^ against ALL reflections. The weighted *R*-factor *wR* and goodness of fit *S* are based on *F*^2^, conventional *R*-factors *R* are based on *F*, with *F* set to zero for negative *F*^2^. The threshold expression of *F*^2^ > σ(*F*^2^) is used only for calculating *R*-factors(gt) *etc*. and is not relevant to the choice of reflections for refinement. *R*-factors based on *F*^2^ are statistically about twice as large as those based on *F*, and *R*- factors based on ALL data will be even larger.

### Fractional atomic coordinates and isotropic or equivalent isotropic displacement parameters (Å^2^)

**Table:**

	*x*	*y*	*z*	*U*_iso_*/*U*_eq_	
Mn1	0.0000	0.0000	0.0000	0.02539 (15)	
N1	−0.0491 (2)	−0.09844 (11)	0.12272 (9)	0.0311 (3)	
N2	−0.1216 (2)	−0.12917 (11)	0.25951 (10)	0.0313 (4)	
N3	0.21756 (19)	0.07932 (11)	0.11619 (9)	0.0305 (3)	
N4	0.3569 (2)	0.14248 (11)	0.26047 (10)	0.0314 (4)	
Cl1	−0.22693 (6)	0.13438 (3)	0.02653 (3)	0.03544 (16)	
C1	0.0739 (2)	−0.16467 (14)	0.18007 (12)	0.0330 (4)	
H1	0.1721	−0.1924	0.1632	0.040*	
C2	0.0323 (3)	−0.18392 (14)	0.26455 (12)	0.0332 (4)	
H2	0.0952	−0.2257	0.3157	0.040*	
C3	−0.1654 (2)	−0.07860 (14)	0.17360 (11)	0.0314 (4)	
H3	−0.2644	−0.0354	0.1526	0.038*	
C4	−0.2131 (3)	−0.11906 (15)	0.33668 (13)	0.0408 (5)	
H4A	−0.2361	−0.1881	0.3587	0.049*	
H4B	−0.3269	−0.0846	0.3107	0.049*	
C5	−0.1034 (2)	−0.05720 (14)	0.42203 (12)	0.0315 (4)	
C6	−0.0835 (3)	0.04960 (15)	0.41478 (12)	0.0377 (4)	
H6	−0.1397	0.0835	0.3570	0.045*	
C7	0.0182 (3)	0.10667 (15)	0.49176 (12)	0.0382 (5)	
H7	0.0296	0.1786	0.4858	0.046*	
C8	0.3906 (3)	0.10316 (15)	0.11778 (13)	0.0379 (4)	
H8	0.4403	0.0942	0.0658	0.046*	
C9	0.4773 (3)	0.14128 (15)	0.20553 (13)	0.0407 (5)	
H9	0.5958	0.1627	0.2252	0.049*	
C10	0.2038 (2)	0.10424 (13)	0.20375 (12)	0.0307 (4)	
H10	0.1002	0.0962	0.2237	0.037*	
C11	0.3886 (3)	0.17165 (15)	0.36308 (12)	0.0385 (5)	
H11A	0.2796	0.2005	0.3731	0.046*	
H11B	0.4790	0.2259	0.3784	0.046*	
C12	0.4485 (2)	0.08073 (13)	0.43225 (11)	0.0287 (4)	
C13	0.5545 (3)	0.10091 (14)	0.52584 (12)	0.0344 (4)	
H13	0.5921	0.1688	0.5437	0.041*	
C14	0.3964 (3)	−0.02103 (14)	0.40819 (12)	0.0347 (4)	
H14	0.3263	−0.0358	0.3458	0.042*	

### Atomic displacement parameters (Å^2^)

**Table:**

	*U*^11^	*U*^22^	*U*^33^	*U*^12^	*U*^13^	*U*^23^
Mn1	0.0305 (2)	0.0259 (2)	0.0201 (2)	0.00112 (14)	0.00755 (16)	0.00075 (13)
N1	0.0381 (8)	0.0307 (8)	0.0249 (7)	−0.0003 (7)	0.0091 (6)	0.0020 (6)
N2	0.0398 (9)	0.0327 (8)	0.0225 (7)	−0.0088 (7)	0.0107 (6)	−0.0040 (6)
N3	0.0309 (8)	0.0342 (8)	0.0262 (7)	0.0028 (6)	0.0072 (6)	0.0002 (6)
N4	0.0355 (9)	0.0307 (8)	0.0246 (7)	0.0000 (6)	0.0019 (6)	0.0038 (6)
Cl1	0.0383 (3)	0.0308 (3)	0.0415 (3)	0.00726 (19)	0.0182 (2)	−0.00047 (18)
C1	0.0372 (10)	0.0295 (9)	0.0317 (9)	0.0010 (8)	0.0083 (8)	−0.0001 (7)
C2	0.0402 (10)	0.0321 (9)	0.0249 (8)	−0.0034 (8)	0.0045 (7)	0.0022 (7)
C3	0.0382 (10)	0.0319 (9)	0.0248 (8)	−0.0013 (8)	0.0097 (7)	−0.0006 (7)
C4	0.0500 (12)	0.0496 (12)	0.0279 (9)	−0.0173 (9)	0.0196 (9)	−0.0101 (8)
C5	0.0376 (10)	0.0340 (9)	0.0257 (8)	−0.0051 (8)	0.0136 (8)	−0.0053 (7)
C6	0.0496 (12)	0.0369 (10)	0.0243 (8)	0.0018 (9)	0.0061 (8)	0.0065 (8)
C7	0.0590 (13)	0.0260 (9)	0.0327 (10)	−0.0029 (9)	0.0175 (9)	0.0003 (7)
C8	0.0360 (11)	0.0464 (11)	0.0329 (10)	0.0021 (9)	0.0119 (8)	0.0010 (8)
C9	0.0305 (10)	0.0439 (11)	0.0443 (11)	−0.0025 (9)	0.0043 (9)	0.0059 (9)
C10	0.0310 (9)	0.0322 (9)	0.0281 (9)	−0.0011 (8)	0.0064 (7)	0.0012 (7)
C11	0.0523 (12)	0.0309 (10)	0.0262 (9)	−0.0010 (9)	0.0000 (8)	−0.0019 (8)
C12	0.0309 (9)	0.0292 (9)	0.0239 (8)	−0.0021 (7)	0.0035 (7)	−0.0009 (7)
C13	0.0425 (10)	0.0264 (9)	0.0292 (9)	−0.0078 (8)	0.0011 (8)	−0.0032 (7)
C14	0.0403 (11)	0.0346 (10)	0.0218 (8)	−0.0039 (8)	−0.0044 (8)	−0.0024 (7)

### Geometric parameters (Å, °)

**Table:**

Mn1—N1^i^	2.2695 (13)	C4—H4A	0.9700
Mn1—N1	2.2695 (13)	C4—H4B	0.9700
Mn1—N3^i^	2.2665 (14)	C5—C6	1.382 (3)
Mn1—N3	2.2665 (14)	C5—C7^ii^	1.384 (2)
Mn1—Cl1^i^	2.5639 (4)	C6—C7	1.378 (3)
Mn1—Cl1	2.5639 (4)	C6—H6	0.9300
N1—C3	1.327 (2)	C7—C5^ii^	1.384 (2)
N1—C1	1.373 (2)	C7—H7	0.9300
N2—C3	1.346 (2)	C8—C9	1.344 (3)
N2—C2	1.373 (2)	C8—H8	0.9300
N2—C4	1.468 (2)	C9—H9	0.9300
N3—C10	1.320 (2)	C10—H10	0.9300
N3—C8	1.375 (2)	C11—C12	1.515 (2)
N4—C10	1.340 (2)	C11—H11A	0.9700
N4—C9	1.373 (2)	C11—H11B	0.9700
N4—C11	1.465 (2)	C12—C14	1.379 (2)
C1—C2	1.351 (2)	C12—C13	1.392 (2)
C1—H1	0.9300	C13—C14^iii^	1.372 (2)
C2—H2	0.9300	C13—H13	0.9300
C3—H3	0.9300	C14—C13^iii^	1.372 (2)
C4—C5	1.509 (2)	C14—H14	0.9300
			
N3^i^—Mn1—N3	180.00 (12)	C5—C4—H4A	109.3
N3^i^—Mn1—N1^i^	86.10 (5)	N2—C4—H4B	109.3
N3—Mn1—N1^i^	93.90 (5)	C5—C4—H4B	109.3
N3^i^—Mn1—N1	93.90 (5)	H4A—C4—H4B	108.0
N3—Mn1—N1	86.10 (5)	C6—C5—C7^ii^	118.81 (15)
N1^i^—Mn1—N1	180.00 (10)	C6—C5—C4	120.56 (16)
N3^i^—Mn1—Cl1^i^	90.07 (4)	C7^ii^—C5—C4	120.62 (16)
N3—Mn1—Cl1^i^	89.93 (4)	C7—C6—C5	121.07 (16)
N1^i^—Mn1—Cl1^i^	89.62 (4)	C7—C6—H6	119.5
N1—Mn1—Cl1^i^	90.38 (4)	C5—C6—H6	119.5
N3^i^—Mn1—Cl1	89.93 (4)	C6—C7—C5^ii^	120.11 (18)
N3—Mn1—Cl1	90.07 (4)	C6—C7—H7	119.9
N1^i^—Mn1—Cl1	90.38 (4)	C5^ii^—C7—H7	119.9
N1—Mn1—Cl1	89.62 (4)	C9—C8—N3	109.95 (16)
Cl1^i^—Mn1—Cl1	180.00 (2)	C9—C8—H8	125.0
C3—N1—C1	105.16 (14)	N3—C8—H8	125.0
C3—N1—Mn1	126.56 (12)	C8—C9—N4	106.61 (16)
C1—N1—Mn1	124.54 (12)	C8—C9—H9	126.7
C3—N2—C2	107.31 (15)	N4—C9—H9	126.7
C3—N2—C4	125.77 (16)	N3—C10—N4	112.02 (16)
C2—N2—C4	126.68 (15)	N3—C10—H10	124.0
C10—N3—C8	104.94 (15)	N4—C10—H10	124.0
C10—N3—Mn1	124.43 (12)	N4—C11—C12	113.24 (15)
C8—N3—Mn1	130.38 (11)	N4—C11—H11A	108.9
C10—N4—C9	106.47 (14)	C12—C11—H11A	108.9
C10—N4—C11	125.48 (16)	N4—C11—H11B	108.9
C9—N4—C11	127.90 (16)	C12—C11—H11B	108.9
C2—C1—N1	110.37 (17)	H11A—C11—H11B	107.7
C2—C1—H1	124.8	C14—C12—C13	118.21 (15)
N1—C1—H1	124.8	C14—C12—C11	122.95 (15)
C1—C2—N2	105.93 (15)	C13—C12—C11	118.78 (15)
C1—C2—H2	127.0	C14^iii^—C13—C12	120.25 (16)
N2—C2—H2	127.0	C14^iii^—C13—H13	119.9
N1—C3—N2	111.22 (16)	C12—C13—H13	119.9
N1—C3—H3	124.4	C13^iii^—C14—C12	121.54 (15)
N2—C3—H3	124.4	C13^iii^—C14—H14	119.2
N2—C4—C5	111.57 (15)	C12—C14—H14	119.2
N2—C4—H4A	109.3		
			
N3^i^—Mn1—N1—C3	−87.09 (14)	C3—N2—C4—C5	−106.8 (2)
N3—Mn1—N1—C3	92.91 (14)	C2—N2—C4—C5	67.0 (2)
Cl1^i^—Mn1—N1—C3	−177.19 (14)	N2—C4—C5—C6	72.4 (2)
Cl1—Mn1—N1—C3	2.81 (14)	N2—C4—C5—C7^ii^	−106.34 (19)
N3^i^—Mn1—N1—C1	118.00 (13)	C7^ii^—C5—C6—C7	−0.4 (3)
N3—Mn1—N1—C1	−62.00 (13)	C4—C5—C6—C7	−179.12 (18)
Cl1^i^—Mn1—N1—C1	27.91 (13)	C5—C6—C7—C5^ii^	0.4 (3)
Cl1—Mn1—N1—C1	−152.09 (13)	C10—N3—C8—C9	0.2 (2)
N1^i^—Mn1—N3—C10	139.92 (14)	Mn1—N3—C8—C9	−174.10 (13)
N1—Mn1—N3—C10	−40.08 (14)	N3—C8—C9—N4	−0.5 (2)
Cl1^i^—Mn1—N3—C10	−130.47 (14)	C10—N4—C9—C8	0.5 (2)
Cl1—Mn1—N3—C10	49.53 (14)	C11—N4—C9—C8	176.26 (17)
N1^i^—Mn1—N3—C8	−46.74 (16)	C8—N3—C10—N4	0.1 (2)
N1—Mn1—N3—C8	133.26 (16)	Mn1—N3—C10—N4	174.89 (11)
Cl1^i^—Mn1—N3—C8	42.87 (15)	C9—N4—C10—N3	−0.4 (2)
Cl1—Mn1—N3—C8	−137.13 (15)	C11—N4—C10—N3	−176.28 (15)
C3—N1—C1—C2	−0.28 (19)	C10—N4—C11—C12	86.3 (2)
Mn1—N1—C1—C2	159.05 (12)	C9—N4—C11—C12	−88.6 (2)
N1—C1—C2—N2	0.6 (2)	N4—C11—C12—C14	−30.7 (2)
C3—N2—C2—C1	−0.67 (19)	N4—C11—C12—C13	152.19 (17)
C4—N2—C2—C1	−175.37 (15)	C14—C12—C13—C14^iii^	−0.5 (3)
C1—N1—C3—N2	−0.16 (19)	C11—C12—C13—C14^iii^	176.75 (18)
Mn1—N1—C3—N2	−158.94 (11)	C13—C12—C14—C13^iii^	0.5 (3)
C2—N2—C3—N1	0.53 (19)	C11—C12—C14—C13^iii^	−176.62 (19)
C4—N2—C3—N1	175.29 (15)		

Symmetry codes: (i) −*x*, −*y*, −*z*; (ii) −*x*, −*y*, −*z*+1; (iii) −*x*+1, −*y*, −*z*+1.

### Hydrogen-bond geometry (Å, °)

**Table:**

Cg is the centroid of the N3,N4,C8–C10 ring.

**Table:**

*D*—H···*A*	*D*—H	H···*A*	*D*···*A*	*D*—H···*A*
C4—H4A···Cg^iv^	0.97	2.65	3.522 (2)	150

Symmetry codes: (iv) −*x*, *y*−1/2, −*z*+1/2.
